# Computational Genomics for Resistome Characterization: Current Advancements and Future Challenges Under a One Health Perspective

**DOI:** 10.3390/antibiotics15080804

**Published:** 2026-08-18

**Authors:** Lenin García Gutiérrez, Alfonso Méndez-Tenorio, Mario Ángel López-Luis, Sandra Alejandra Ávila-Huerta, Gloria León-Ávila, Santiago R. Castaño-Valencia, Gabriela Ibáñez-Cervantes

**Affiliations:** 1 Departamento de Zoología, Escuela Nacional de Ciencias Biológicas, Instituto Politécnico Nacional, Mexico City 11340, Mexico; 2 Laboratorio de Biotecnología y Bioinformática Genómica, Departamento de Bioquímica, Escuela Nacional de Ciencias Biológicas, Instituto Politécnico Nacional, Mexico City 11340, Mexico; 3 Laboratorio de Helmintología, Departamento de Parasitología, Escuela Nacional de Ciencias Biológicas, Instituto Politécnico Nacional, Mexico City 11340, Mexico; 4 Laboratorio de Herpetología y Toxinología, Departamento de Ciencias Fisiológicas, Departamento de Ciencias de la Salud, Universidad del Valle, Cali 760031, Colombia; 5 Laboratorio de Microbiología y Biología Molecular, Departamento de Desarrollo de Tecnologías, Centro Interdisciplinario de Ciencias Marinas, Instituto Politécnico Nacional, C. Instituto Politécnico Nacional s/n, La Paz 23096, Mexico

**Keywords:** resistome, metagenomics, antimicrobial resistance, bioinformatics, machine learning, One Health

## Abstract

The resistome, defined as the complete set of antibiotic resistance genes (ARGs) present in the microbiota of a given environment, is a critical component for understanding the evolutionary dynamics of antimicrobial resistance (AMR) and its impact on human, animal, and environmental health. This review summarizes current methods and technological advances and offers a forward-looking perspective on resistome research. A systematic literature search was conducted. References on short-read and long-read sequencing, amplicon sequencing, shotgun metagenomics, and multi-omics integration were included, as were bioinformatics tools for the detection, quantification, and annotation of ARGs. The results indicate that next-generation sequencing (NGS) technologies have significantly improved the characterization of ARGs across ecosystems, enabling high-resolution microbial profiling and the discovery of new variants. Furthermore, integrating multi-omics approaches with computational tools improves data accuracy, reduces analysis and reporting times, and facilitates the development of predictive models. However, significant challenges remain, which will be key to strengthening epidemiological surveillance under the One Health approach.

## 1. Introduction

Antimicrobial resistance (AMR) is a growing threat to global public health and is increasingly recognized as a leading cause of mortality worldwide. AMR represents one of the most pressing challenges to global health, threatening the effectiveness of existing antimicrobial therapies and contributing substantially to morbidity and mortality worldwide [1,2]. The emergence and dissemination of resistance determinants across diverse microbial communities have become increasingly complex, requiring integrative approaches capable of resolving the diversity, dynamics and ecological drivers of the resistome. Recent advances in next-generation sequencing (NGS) have transformed metagenomic research by enabling the comprehensive characterization of microbial communities. In parallel, the development of scalable bioinformatics pipelines has facilitated the analysis of large-scale metagenomic datasets across human, animal and environmental reservoirs [3]. These approaches have provided deeper insights into how resistance genes shape bacterial biology, revealing structural details of key molecular processes, facilitating the identification of novel resistance gene families, and uncovering interactions among different resistance mechanisms. Collectively, these tools are critical for the development of next-generation antimicrobial strategies and therapies. These advances have expanded our understanding of the genetic architecture and evolutionary dynamics of resistance, facilitating the discovery of novel ARG families, the identification of mobile resistance elements, and the elucidation of interactions among co-occurring resistance mechanisms. Collectively, these advances provide a critical foundation for developing predictive surveillance strategies and guiding the discovery of next-generation antimicrobial interventions.

## 2. Methodology

This review was conducted in accordance with the Preferred Reporting Items for Systematic Reviews and Meta-Analyses (PRISMA 2020) guidelines to ensure a transparent and reproducible literature selection process. A comprehensive literature search was conducted across PubMed, Scopus (Elsevier), and the National Center for Biotechnology Information (NCBI) databases, supplemented by bioRxiv for relevant preprint studies, to identify studies related to next-generation sequencing (NGS), metagenomics, microbiome research, antimicrobial resistance (AMR), resistome surveillance, and the One Health framework. Search strategies combined controlled vocabulary and free-text terms, including “next-generation sequencing”, “metagenomics”, “microbiome”, “resistome”, “antimicrobial resistance”, “antibiotic resistance genes”, “bioinformatics”, and “One Health”.

A literature search covered the period from 2005 to 2026. A total of 262 references were initially identified through database searches and computational repositories. All sources were exported to reference management software, where duplicate records were identified and removed before screening. Titles and abstracts were independently evaluated for relevance to the objectives of this review, followed by full-text assessment of potentially eligible articles. Studies were considered eligible if they reported advances in sequencing technologies, bioinformatics methodologies, computational tools, resistome characterization, antimicrobial resistance surveillance, or One Health applications. Conference abstracts, editorials, non-English publications, duplicate reports, and studies outside the scope of sequencing-based resistome surveillance were excluded. In addition to peer-reviewed publications, GitHub repositories (https://github.com/ (accessed on 11 August 2026)) and publicly available bioinformatics resources were systematically screened to identify widely used software tools, computational pipelines, and analytical frameworks relevant to metagenomic and resistome analyses. Following the eligibility assessment, 182 studies were included and formed the evidence base for this review. The final body of evidence was selected based on methodological relevance to the objectives of this review. The complete literature selection process included record identification, screening, eligibility assessment, and study inclusion. These studies collectively demonstrate how next-generation sequencing (NGS) technologies are advancing microbiome-based diagnostics, resistome characterization, and public health applications.

## 3. Sequencing Methods and Omics Approaches for the Study of the Resistome

Studies of the microbiome and its resistome have benefited greatly from technological advances in next-generation sequencing (NGS). These advances have enabled characterization of complex microbial communities at multiple molecular levels—genomic, transcriptomic, proteomic, and metabolomic—and determination of their functional profiles [4].

**Figure 1:**
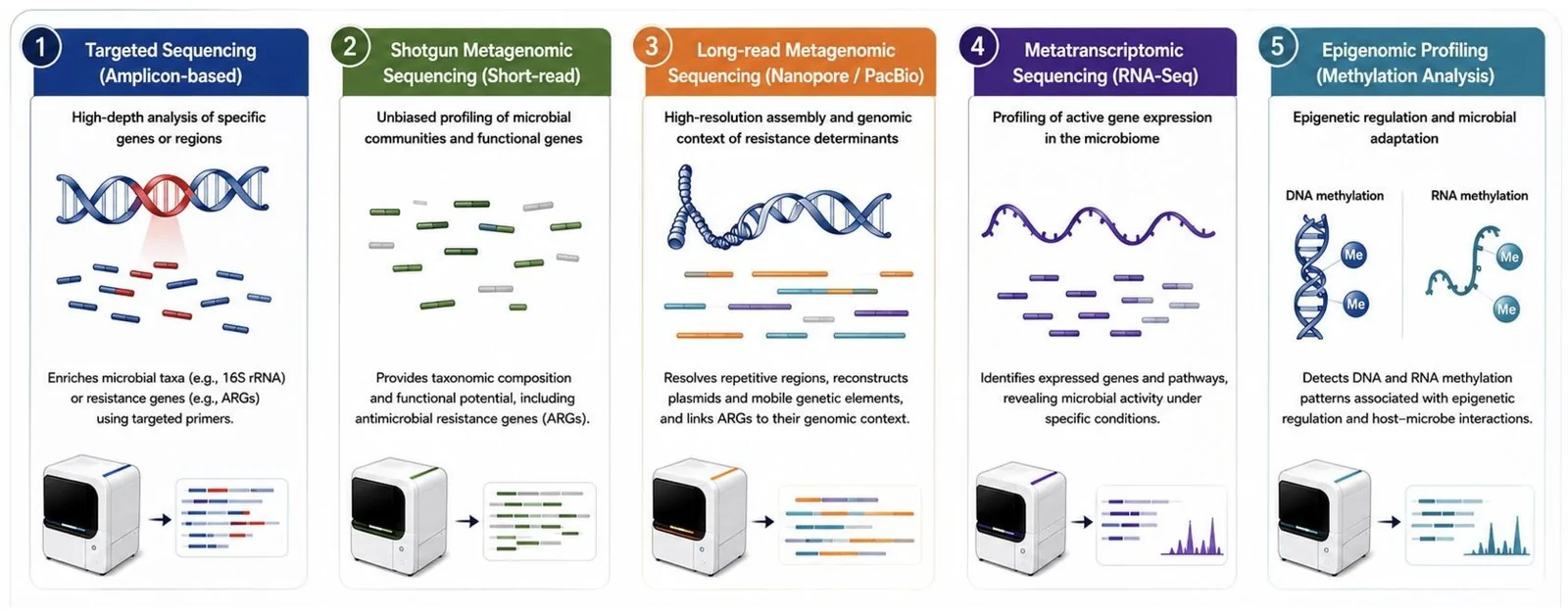
Sequencing strategies for comprehensive characterization of the microbiome and antimicrobial resistome. (1) Targeted sequencing uses specific primers to amplify and sequence predefined genes or regions, enabling high-depth analysis of microbial taxa (16S rRNA) or antimicrobial resistance genes (ARGs); (2) Shotgun metagenomic sequencing with short-read platforms (Illumina) provides an unbiased view of microbial community composition and functional gene context; however, short reads are limited in resolving repetitive regions and in linking ARGs to mobile genetic elements. (3) Long-read metagenomic sequencing (Oxford Nanopore Technologies, PacBio) generates long, continuous reads that improve assembly quality, resolve repetitive sequences, reconstruct plasmids and mobile genetic elements, and accurately associate ARGs with their genomic context. (4) Metatranscriptomic sequencing (RNA-Seq) captures and quantifies actively expressed microbial genes, providing insights into functional and microbial responses under physiological or pathological conditions. (5) Epigenomic profiling analyzes DNA and RNA methylation patterns using single-molecule sequencing, revealing epigenetic regulation involved in microbial adaptation and host–microbiome interactions.

Based on these technologies, five main strategies have been developed, with a forward-looking focus on incorporating epigenomic processes, as illustrated in Figure 1. These strategies enable high-throughput analysis of DNA and RNA, optimize turnaround times, reduce costs in less complex diagnostic applications, and support large-scale, parallel data processing. They are categorized as amplicon-based targeted sequencing, short-read shotgun metagenomics, long-read metagenomics, metatranscriptomics (RNA-Seq), and epigenomic profiling [5]. Currently, epigenomic profiling and methylation analysis are increasingly integrated, opening new avenues for investigating complex regulatory mechanisms and advanced structural modifications.

### 3.1. Next-Generation Sequencing (NGS)

Next-Generation Sequencing (NGS) encompasses a set of high-throughput technologies that enable massive parallel sequencing of DNA, facilitating the identification and characterization of microorganisms—including uncultivable ones—and the prediction of their biological function [6]. Depending on the sequencing strategy employed, NGS can support either taxonomic profiling of microbial communities or functional profiling.

Among the various approaches, amplicon sequencing is one of the most widely used strategies for microbiome analysis, based on the amplification of marker genes such as 16S rRNA (V1–V2, V3–V4, V4, V5–V7, and V7–V9) in bacteria, 18S rRNA (V4 and V4–V6), and the fungal internal transcribed spacer (ITS) regions (ITS1 and ITS2) [7]. Its application to samples with low biomass allows for an expansion of the scope of microbiological studies. However, despite the intragenic redundancy of 16S rRNA, the selection of variable regions, and the heterogeneity of the sequences, the choice of primers and the depth of sequencing influence the quality of the data obtained and may introduce bases [8].

In contrast, second- and third-generation sequencing methods, including short-read (Illumina) and long-read (PacBio and Oxford Nanopore Technologies) platforms, each have distinct strengths. Short reads can identify antibiotic resistance genes (ARGs) but often cannot reliably establish their physical linkage to plasmids or other mobile genetic elements. Long-read sequencing provides extended genomic context and improves resolution of repetitive regions, plasmids, and mobile genetic elements associated with ARGs. PacBio HiFi sequencing combines long reads with high base accuracy, whereas Oxford Nanopore Technologies generates long to ultra-long reads with continuously improving accuracy, depending on sequencing chemistry and base-calling approaches. This capability is critical for determining the genomic context and potential mobility of ARGs, thereby improving our understanding of the mechanisms underlying the dissemination of antimicrobial resistance [9,10].

The integration of artificial intelligence (AI) tools, with a focus on machine learning and deep learning, is rapidly transforming sequencing technologies. The Illumina platform implements software-accelerated solutions such as DRAGEN (Dynamic Read Analysis for GENomics), which significantly improve the speed and accuracy of alignment, variant calling, and metagenomic analysis [11]. In addition to long-read technologies, AI has been instrumental in optimizing processes such as base calling through advanced models (Dorado), reducing error rates, and improving the resolution of entire genomic regions. Recent studies have shown that these approaches also enable the integration of raw sequencing data to detect epigenetic modifications in real time, thereby expanding the analytical capabilities of these platforms [12]. In this context, third-generation technologies offer greater potential for clinical, environmental, and epidemiological applications owing to their ability to generate long sequencing reads, accurately characterize a broader range of microorganisms—including parasites, viruses, and fungi—and simultaneously enable comprehensive analyses of the resistome, mobilome, and other functional genomics features within integrated multi-omics frameworks [13].

This advancement has also shifted the analytical approach, requiring a robust computational infrastructure that includes high-capacity storage systems, high-performance computing, and specialized bioinformatics tools capable of managing, analyzing, and processing large-scale data. In this context, the growing demand for analytical efficiency, automation, and multi-omics data integration is driving the development of next-generation bioinformatics frameworks [14,15]. This new phase will natively implement artificial intelligence and hybrid computing architectures for real-time analysis, not only generating data but also providing immediate interpretation and automated decision-making [16]. Next-generation bioinformatics frameworks will need to address limitations in data storage, cross-disciplinary multi-omics integration, and databases, while providing platforms that streamline, automate, and enable rapid, high-specificity analyses with direct applications in clinical diagnosis, environmental monitoring, and epidemiological surveillance.

### 3.2. Metagenomic Strategies for Characterization

Metagenomics is an essential approach for understanding the resistome, serving as a functional tool for analyzing genetic material from the microbial community without the need for culture. This approach enables characterization of the microbiome’s taxonomic and functional diversity and detection and quantification of resistance genes and other functional profiles [17,18]. Although these molecular approaches are often discussed together in microbiome studies, they serve distinct analytical purposes and should not be considered interchangeable. Amplicon sequencing of phylogenetic marker genes (16S rRNA, 18S rRNA, and ITS) is designed for taxonomic profiling and does not directly detect or quantify antimicrobial resistance genes (ARGs). In contrast, shotgun metagenomics enable simultaneous characterization of microbial community composition, ARGs, and their genomic context, whereas quantitative PCR (qPCR) is a targeted approach for the detection and quantification of predefined ARGs rather than a metagenomic sequencing strategy. The principal molecular approaches currently used for microbiome profiling and resistome characterization are summarized in Table 1.

#### 3.2.1. Quantitative PCR (qPCR)

qPCR is a highly sensitive and specific approach widely validated for quantifying the absolute or relative abundance of known antibiotic resistance genes (ARGs) across diverse conditions [19]. Unlike metagenomic sequencing, qPCR does not generate sequence data or provide genome-wide characterization of microbial communities. Instead, it quantifies the absolute or relative abundance of previously characterized ARGs using gene-specific primer sets. Although qPCR offers high analytical sensitivity, specificity, and rapid turnaround, its reliance on predefined primer sets and reference sequences limits its ability to detect novel ARGs, previously uncharacterized sequence variants, or the genomic context of resistance determinants, including their association with mobile genetic elements (MGEs). Consequently, qPCR should be regarded as a complementary targeted validation and surveillance tool rather than a metagenomic sequencing approach.

#### 3.2.2. Amplicon Sequencing

Amplicon sequencing uses phylogenetic marker genes such as the 16S ribosomal DNA (16S rDNA) gene in prokaryotes and 18S rDNA or internal transcribed spacers (ITS) in eukaryotes, with the 16S gene being the most widely used [20]. This approach enables high-throughput profiling across large sample cohorts; however, its taxonomic resolution is typically limited to the genus level due to primer bias, the targeted variable region, PCR cycle count, and variation in 16S rRNA gene copy number [21,22]. Amplicon sequencing targets phylogenetic marker genes and cannot directly identify or quantify antibiotic resistance genes (ARGS) or mobile genetic elements (MGEs). Nevertheless, establishing an accurate taxonomic profile remains essential, as it provides the foundational microbial community framework required to contextualize, model, and interpret resistome dynamics within the sample.

#### 3.2.3. Shotgun Metagenomics

Shotgun metagenomics enables untargeted analysis of the entire genetic context of a sample, including both cultivable and non-culturable organisms [23]. It has become the most robust strategy for characterizing the system, as it allows for the detection of both known ARGs and new variants. Its main limitations include contamination with host DNA, which reduces sequencing depth, and the incomplete assembly of small contigs, making it difficult to reconstruct transposons, plasmids, and other mobile genetic elements (MGEs) that carry ARGs [24,25]. Furthermore, in low-coverage samples, the assay’s sensitivity decreases significantly; this limitation has been mitigated by new sequencing technologies.

#### 3.2.4. Functional Metagenomics

It is a definitive tool that introduces heterologous expression systems to move beyond future in silico predictions and validate the biological activity of genetic determinants in complex ecosystems [26]. Unlike shotgun metagenomics, functional screening enables the identification of antimicrobial resistance genes (ARGs) along with their protein structures, thereby going beyond conventional annotation algorithms. Constructing cloning libraries in expression vectors and performing phenotypic selection in heterologous hosts (e.g., *E. coli*) enable this methodology to reveal reservoirs in microbiomes that standard genomic analyses cannot detect [27,28]. However, placing genes outside their native context and under a substitute expression system alters the framework of their original cellular nature and physiology. 

Within the framework of the One Health approach, functional metagenomics not only confirms the efficacy of ARGs but also enables characterization of the hidden resistome, its potential for horizontal transfer, and the mechanisms of bacterial persistence against new antibiotics [29].

### 3.3. Types of Readings

Among metagenomic sequencing strategies, the read type is the primary determinant of the analysis’s resolution and scope. In this regard, current technologies are primarily classified as short-read and long-read sequencing, each with specific advantages and limitations for studying the microbiome and resistome.

#### 3.3.1. Short Reads

Short-read sequencing, as used by Illumina platforms, generates reads of 75 to 300 bp in either single-end or paired-end configurations (e.g., 2 × 150 bp or 2 × 300 bp, where each value represents the length of an individual read). Its use in metagenomic studies provides a precise approach, with error rates generally below 1% (>99% accuracy) and a low cost per sequenced base, enabling taxonomic and functional profiling of microbial communities and accurate detection of antimicrobial resistance genes (ARGs) [30]. However, its relatively short read length limits complete genome assembly and prevents reliable reconstruction of repetitive genomic regions or the physical linkage of ARGs to plasmids, integrons, transposons, and other mobile genetic elements (MGEs) [31].

#### 3.3.2. Long Reads

Long-read sequencing, implemented on platforms such as Pacific Biosciences and Oxford Nanopore Technologies, yields DNA fragments that typically exceed 10 kb [32]. In the case of PacBio, HiFi (High-Fidelity) sequencing is based on circular consensus sequencing (CCS), in which the same DNA molecule is sequenced multiple times to generate highly accurate consensus reads. HiFi reads typically combine long read lengths (approximately 15–25 kb on average) with high accuracy (>99.9%), though overall sequencing performance depends on library preparation, sequencing chemistry, and platform configuration. Oxford Nanopore Technologies sequencing detects changes in ionic current as individual DNA or RNA molecules pass through a biological nanopore, enabling real-time sequencing [33]. Depending on the library preparation protocol and sample input, native DNA can be sequenced using amplification-free workflows or PCR-based approaches when material is limited. For transcriptomic applications, the platform supports both direct RNA sequencing—analyzing native RNA molecules—and cDNA sequencing, in which RNA is reverse-transcribed prior to sequencing. Oxford Nanopore Technologies generates long to ultra-long reads, frequently exceeding 100 kb under optimized conditions, which facilitates de novo genome assembly and improves the resolution of repetitive, structurally complex regions and mobile genetic elements (MGEs) that remain challenging for short-read platforms. In terms of accuracy, long reads have historically had higher error rates (5–10%); however, recent advances in base-calling algorithms and sequencing chemistry have significantly increased accuracy, reaching values comparable to or exceeding 99% under optimized conditions [34,35]. These advancements are driven by continuous improvements in sequencing chemistry, pore design, and the development of artificial intelligence-based algorithms. Future development is designed to focus on solid-state silicon or polymer nanopores that allow the passage of a single nucleotide at a time, which could significantly improve resolution, accuracy, and integration into microelectronic platforms [33,36].

These features enable more accurate detection of the genomic context of antibiotic resistance genes (ARGs), including their association with mobile genetic elements such as plasmids, transposons, and integrons, and the identification of structural variants. Long-read sequencing facilitates functional characterization by determining transcript isoforms and analyzing epigenetic modifications, providing key insights into how resistance affects cellular physiology [37,38].

### 3.4. Multi-Omics Integration

The transition from metagenomics to systems biology is underpinned by the complex network of interactions among the microbiome, resistome, and host [39]. The potential of multi-omics integration lies in its ability to consolidate robust molecular signatures that surpass the resolution of isolated taxonomic biomarkers. Metagenomics identifies genetic potential; incorporating transcriptomic, proteomic, and metabolomic data filters out biological noise and extracts activity patterns consistent across various pathologies [40]. These multi-omics signatures reveal shared mechanisms (metabolic pathways, inflammation, or efflux pump expression) across the spectrum of chronic and infectious diseases [41,42,43]. The One Health concept benefits from genomic surveillance, which not only detects risks but also predicts functional impact through comprehensive signatures.

## 4. Databases and Bioinformatics Tools for Resistome Analysis

Resistome analysis depends on the quality, curation level, and conceptual structure of antibiotic resistance gene (ARG) databases, as well as on the computational strategy used to detect ARGs [44]. Databases have evolved from static repositories to dynamic, comprehensive platforms with advanced computational support and are publicly available through NCBI. These tools enable the generation of large volumes of biological sequencing data, facilitating characterization of ARG presence, abundance, and diversity in both clinical and environmental microbiomes [45].

These databases can generally be classified as general-purpose platforms and specific repositories. Among the general-purpose databases, CARD (Comprehensive Antibiotic Resistance Database) is one of the most widely used resources [46]. CARD is distinguished by its manual curation and its implementation of the Antibiotic Resistance Ontology (ARO), which provides a structured framework for classifying resistance genes, mechanisms, and phenotypes [47]. This architecture allows for the integration of experimental evidence, such as computational predictions, thereby enhancing analysis and contextual understanding. It also integrates heterogeneous data sources, including computational predictions, thereby improving analytical accuracy and biological interpretation. However, its emphasis on validated sequences may limit its ability to detect variants.

ResFinder was developed primarily for clinical applications, enabling the identification of acquired resistance genes by aligning sequences to reference sequences [48,49]. Its ease of use and accessibility via web platforms have made it a standard tool for epidemiological surveillance. However, its limitations in assessing sequence similarity restrict its ability to detect hypervariable genes.

AMRFinderPlus, developed by NCBI, takes a comprehensive approach: it considers not only genes but also point mutations and intrinsic resistance determinants. The goal is to optimize accuracy and reduce false positives, making it suitable for clinical and public health applications. However, its coverage may be less comprehensive in complex environmental settings [50].

The ARG-ANNOT (antibiotic Resistance Gene-ANNOTation) database provides a resource for the systematic identification of acquired resistance genes using sequence-similarity-based approaches derived from GenBank. ARG-ANNOT organizes resistance determinants into functional categories, facilitating their integration into bioinformatics pipelines for genomic and metagenomic analysis. It is used in comparative studies and in the characterization of the bacterial resistome, with alignment tools. This approach relies on sequence data; therefore, its capabilities are limited compared to more recent databases and approaches based on probabilistic models or machine learning [51].

In the metagenomic field, MEGARes was designed for shotgun metagenomics. With its hierarchical structure, it enables the classification of resistance determinants not only to antibiotics but also to biocides and heavy metals, thereby broadening our understanding of selective pressure across various ecosystems. This characterization proves relevant for environmental studies through the use of specialized pipelines [52].

On the other hand, FARME introduces an approach based on functional metagenomics that enables the identification of ARGs by biological activity and reveals the considerable diversity of previously described resistance genes, especially in environments with high microbial diversity [53]. Interpreting these results within standardized comparative frameworks remains challenging.

Tools such as ShortBRED and SRST2 have significantly improved the detection and quantification of antimicrobial resistance genes (ARGs). ShortBRED uses markers specific to protein families, improving accuracy in complex metagenomic data and reducing ambiguity compared with conventional alignments. Meanwhile, SRST2 enables rapid identification of resistance genes directly from short reads, making it useful in clinical and epidemiological surveillance settings [54,55].

Current developments in artificial intelligence-based tools are transforming these fields. Deep learning models, such as DeepARG, enable the identification of functional patterns in highly divergent sequences, overcoming the limitations of traditional alignment-based approaches [56]. Integrating multi-omics data (metagenomics, metatranscriptomics, proteomics, and metabolomics) not only enables the detection of ARGs but also allows for the assessment of their expression and functional relevance in specific ecological contexts.

In addition to DeepARG, other deep learning tools, such as HMD-ARG and ONN4ARG, incorporate multitasking architectures and ontological knowledge to improve hierarchical classification of resistance genes, handle highly divergent sequences, and overcome the limitations of traditional alignment-based methods [57]. More general models, such as ProtCNN and DeepMicrobes, demonstrate the potential of deep learning to capture complex functional patterns in biological data, pointing toward more robust and integrated predictive systems [58].

### 4.1. Limitations of Databases for Identifying Resistance Genes

Despite significant advances in developing databases for detecting antimicrobial resistance genes (ARGs), major limitations persist that affect accuracy, timeliness, and the identification of new variants [59]. These limitations are especially relevant to resistome analysis, given the rapid genetic evolution and microbial diversity, which pose a constant challenge.

One of the main problems is inadequate database curation. The continuous emergence of new resistance mechanisms, such as *blaNDM-1*-type carbapenemases or *mcr-1* colistinases, requires frequent updates to databases of emerging genes and, consequently, results in an incomplete characterization of the resistome. These studies [60,61,62] have highlighted the rapid emergence of new genetic determinants, which exceeds the capacity to integrate them into current repositories.

Another limitation is the generation of erroneous predictions, including both false positives and false negatives. Identifying homologs of resistance genes does not necessarily imply phenotypic functionality, which can overestimate the presence of resistance. Conversely, point mutations in essential genes can confer resistance, yet these may not be detected by approaches based on sequence similarity. This is a well-documented issue [63,64,65,66], highlighting the need to integrate functional and structural data into bioinformatics analyses. Inconsistent nomenclature across databases poses a significant obstacle, as each repository uses its own classification system. This lack of standards limits tool interoperability and complicates data integration in comparative studies.

Finally, incomplete coverage of the resistome remains a critical challenge because the enormous microbial genetic diversity—particularly in natural environments and among more complex microbes with a high proportion of uncultivable species—limits our ability to detect existing ARGs. The literature [67,68] suggests that a considerable fraction of the global resistome remains uncharacterized, underscoring the need to develop a comprehensive approach that integrates metagenomics with artificial intelligence. These limitations highlight that current databases are fundamental tools for studying the microbiome and its ecosystem and represent an area of opportunity for comprehensive health based on the One Health concept.

### 4.2. Bioinformatic Tools and Pipelines for Resistome Analysis

**Figure 2:**
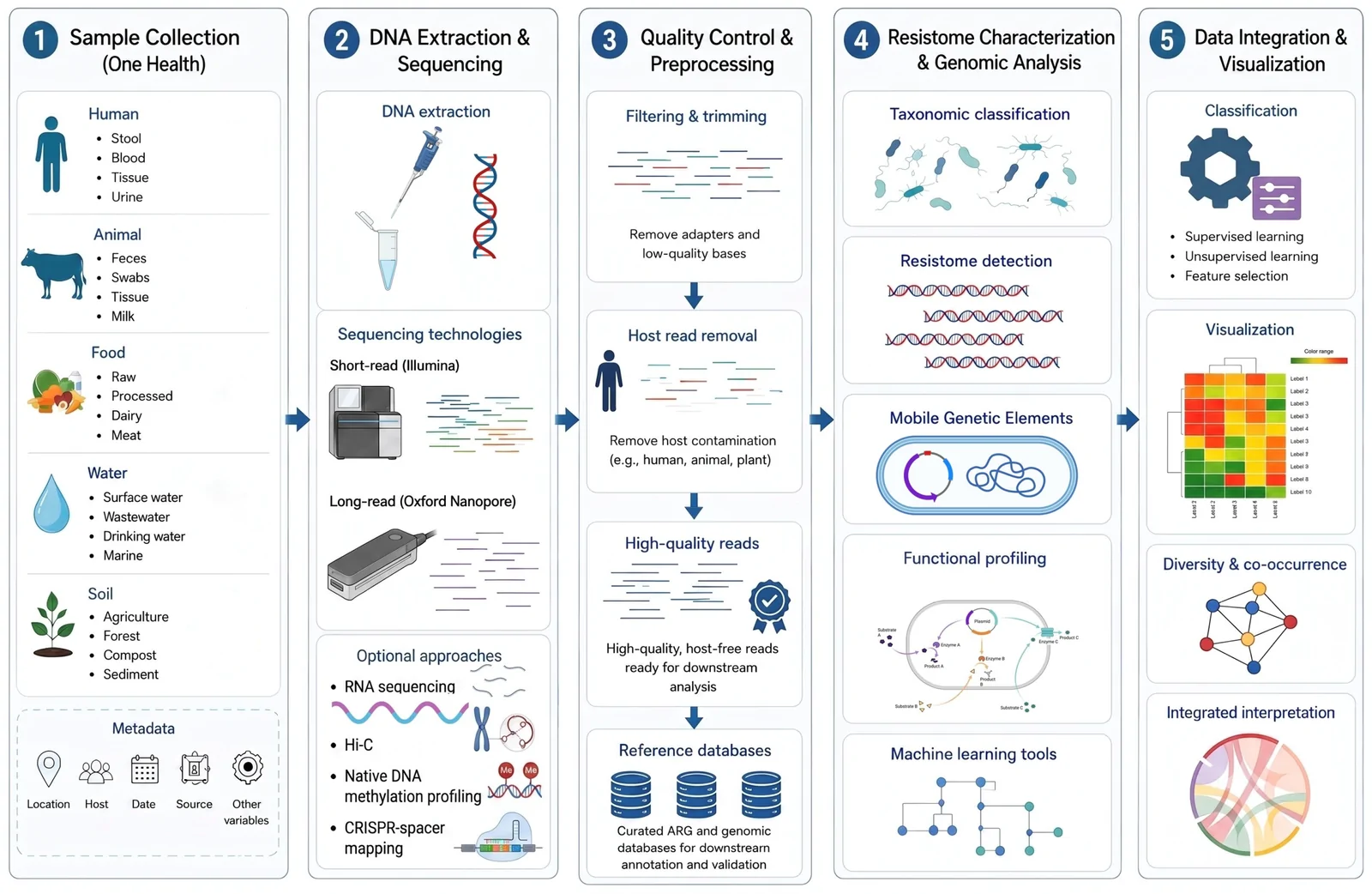
Integrated bioinformatics workflow for One Health shotgun metagenomic resistome profiling. Schematic overview of the analytical pipeline, structured into five core stages: (1) Sample collection and standardized metadata acquisition across human, animal, food, and environmental domains. (2) High-molecular-weight DNA extraction and hybrid sequencing strategies (short- and long-read platforms), complemented by functional genomic extensions. (3) Bioinformatic preprocessing, quality control, and dehosting of human/host DNA. (4) Multi-tiered resistome annotation, including ARG identification, MGE linkage, taxonomic assignment, and machine-learning frameworks. (5) Downstream data integration, statistical ecology, co-occurrence network analysis, and risk assessment models. Representative bioinformatic tools and reference databases are indicated for each stage.

Resistome analysis based on current metagenomic data is being driven by the exponential growth of sequencing data, the development of new long-read technologies, and integration with artificial intelligence. Currently, bioinformatics pipelines range from quality control and classification to identification of resistance genes, functional interpretation modules, risk classification, and predictive analysis, as shown in Figure 2 below [69,70]. Rather than representing a single fixed-operations software, Figure 2 serves as a generalized reference framework that compiles widely accepted best-practice analytical steps and open-source tools. Critical evaluation of these approaches highlights key methodological trade-offs. While read-based ARG detection strategies (e.g., using Kraken2 or alignment tools against databases like CARD or ResFinder) enable rapid profiling and low computational overhead, they often lack genomic context and struggle to differentiate between chromosomal mutations and plasmid-borne resistance determinants. Conversely, assembly-based approaches (e.g., SPAdes, MEGAHIT) reconstruct contigs to link ARGs with mobile genetic elements (MGEs) and host taxonomy but require significantly higher sequencing depth and computational power and often produce fragmented assemblies in hypercomplex environmental matrices. Hybrid sequencing, combining short-read accuracy (Illumina) with long-read structural resolution (Oxford Nanopore Technologies/PacBio), substantially overcomes assembly bottlenecks, though standardization and error-rate mitigation in long reads remain ongoing challenges. Furthermore, database dependence poses an inherent limitation across all steps, as novel or highly divergent ARGs may be missed by current reference repositories.

Characterizing the resistome via high throughput metagenomic sequencing within the “One Health” framework relies on a bioinformatics workflow structured into five stages (Figure 2): standardized sampling and metadata acquisition (Stage 1), hybrid sequencing strategies (Stage 2), quality control and host sequence removal (Stage 3), multilevel annotation (Stage 4), and predictive and ecological risk modeling (Stage 5). Resistome characterization across these stages depends heavily on the strategic selection of reference databases and annotation frameworks (Figure 2, boxes 4 and 5). Beyond traditional antibiotic resistance gene (ARG) repositories, assessing co-selection factors within the “One Health” paradigm also requires characterizing resistance to biocides and heavy metals, mobile genetic elements (MGEs), and functional metabolic pathways. To facilitate tool selection for this analytical workflow, Table 1 systematically compares key reference databases, annotation tools, and machine learning frameworks based on their scope, curation methodology, operational strengths, limitations, and update frequency.

As summarized in Table 2, the choice of reference database and analytical tool entails inherent methodological trade-offs that directly affect resistome interpretation. Clinically focused databases such as ResFinder and AMRFinderPlus offer high specificity for acquired determinants in human pathogens, whereas broader, ontology-driven repositories like CARD and MEGARes provide deeper mechanistic coverage across complex environmental and One Health matrices. Furthermore, recent benchmarking shows that alignment-based ARG detection is constrained by reference database completeness and often misses novel or highly divergent resistance variants. To address this bottleneck, protein language models (PLM-ARG) and deep learning frameworks (DeepARG) have emerged as powerful complementary tools for discovering resistance genes, though they require rigorous experimental validation. Finally, incorporating experimental and computational host-attribution methods—such as native DNA methylation profiling, Hi-C proximity ligation, and CRISPR-spacer mapping—provides promising avenues to address current analytical bottlenecks, helping bridge the gap between simple gene cataloging and the evaluation of the real-world dissemination risk of mobile ARGs within host–microbiome networks.

The choice of a pipeline depends on multiple factors, including the type of data, sequencing depth, microbiome complexity, and the study objective. Pipelines can range from descriptive characterization to real-time epidemiological surveillance or risk assessment under the One Health approach [95]. In this context, current pipelines tend to be modular, reproducible, and scalable thanks to the use of new environments such as Nextflow or Snakemake, the use of scikit-learn, PyTorch, and TensorFlow, or by running them on cloud computing infrastructures [96,97].

#### 4.2.1. Quality Control and Preprocessing

Quality control remains the critical step in ensuring the reliability of the analysis. Traditional tools such as FastQC and Trimmomatic are still widely used for quality assessment, adapter removal, and read filtering. For long reads, specialized tools such as NanoPlot help optimize this process [98,99,100].

Its clinical applications enable the removal of host sequences to eliminate and prevent bias in microbial detection. This process has been refined over time and is now further optimized with faster and more accurate machine-learning algorithms, enabling more precise distinction between human and microbial sequences [101].

#### 4.2.2. Assembly, Mapping and New Hybrid Strategies

Current strategies combine traditional approaches with hybrid methodologies. Sequences can be analyzed directly by aligning them to ARG databases or assembled into longer contigs or MAGs to provide genomic context [102].

Alignment tools such as BLAST and DIAMOND remain fundamental to homology-based search strategies and have been integrated into modern annotation pipelines [103]. For metagenomic assembly, short-read sequencing tools such as MEGAHIT and metaSPAdes have been optimized for complex datasets. Long-read sequencing has further driven the development of specialized metagenomic assemblers, such as MetaFlye, which are tailored to navigate complex community structures, resolve highly repetitive regions, and reconstruct complete microbial genomes alongside mobile genetic elements (MGEs). While hybrid assemblers like Unicycler were originally designed for short- and long-read integration in bacterial isolates [104,105,106,107], dedicated hybrid metagenomics tools (OPERA-MS or hybridSPAdes) are increasingly used to resolve complex metagenomic structures and complete plasmid sequences across diverse microbial communities.

Long-read sequencing (Oxford Nanopore Technologies and PacBio) has revolutionized the analysis of the system by enabling the direct identification of colocalization of ARGs with plasmids, transposons, and other mobile elements, thereby providing critical information on the potential for horizontal transfer [108].

#### 4.2.3. ARG Identification and Profiling

To enable scalable and reproducible resistome characterization, several automated bioinformatics platforms have been developed. ABRicate (https://github.com/tseemann/abricate (accessed on 11 August 2026)) uses pre-indexed databases—CARD, ARG-ANNOT, ResFinder, MEGARes, NCBI AMRFinderPlus, EcOH, PlasmidFinder, Ecoli VF and VFDB, VICTORS, BacMet, and UPEC/ExPEC VF—to rapidly annotate resistance determinants, virulence factors, and plasmid incompatibility groups. While highly efficient for broad screening of assembled sequences, its diagnostic accuracy depends on regular local database updates and on user-defined coverage and identity thresholds.

Complementing assembly-based screening, cloud- and desktop-based platforms such as EPI2ME (developed by Oxford Nanopore Technologies) enable real-time analysis directly during sequencing runs. EPI2ME provides containerized, automated workflows (Nextflow-based EPI2ME Workflows) that integrate real-time basecalling, taxonomic classification (via tools like Kraken2 or Centrifuge), and ARG profiling against databases such as CARD. Although these integrated workflows streamline clinical, epidemiological, and field-based surveillance by drastically reducing turnaround time, they offer limited algorithm customization and transparency compared to custom open-source pipelines [109,110].

Among other platforms is Galaxy, which provides accessible web-based environments that let users run complex pipelines without advanced programming and integrate multiple tools and databases into reproducible workflows [111]. Similarly, KBase (DOE Systems Biology Knowledgebase) enables the integration and analysis of multi-omic data, thereby expanding the functional context of the resistome [112].

Overall, the evolution of these platforms reflects a shift toward increasingly integrated, automated, and scalable bioinformatics environments. These platforms can incorporate multiple databases, analytical tools, and advanced computational approaches, and their integration is essential for improving the sensitivity, accuracy, and reproducibility of analyses and for meeting the growing demand for global surveillance of antimicrobial resistance.

## 5. Determinants Shaping the Resistome and Its Dissemination

**Figure 3:**
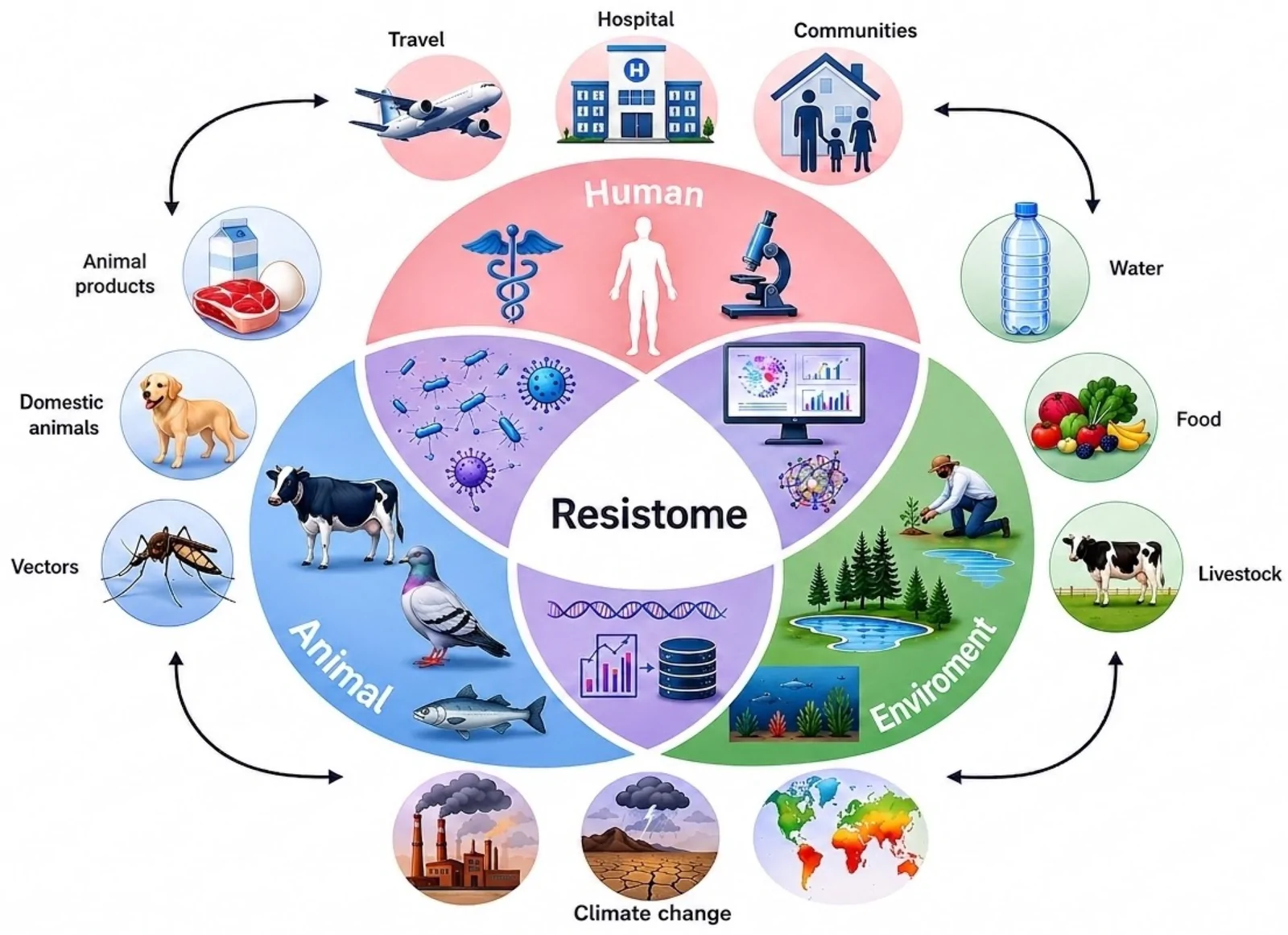
One Health framework for antimicrobial resistance gene (ARG) transmission and ecosystem connectivity. The central Venn diagram depicts the convergence of human (clinical and community), animal (livestock and wildlife), and environmental (aquatic and terrestrial) reservoirs, driving resistome dynamics through integrated genomic and bioinformatic analyses. Peripheral nodes highlight key transmission vectors and environmental drivers, including food production systems, water bodies, trade, travel, vector-borne routes, and climate change. Black double-headed arrows denote the continuous, bidirectional exchange of resistant bacteria and ARGs across ecological interfaces. Cross-sectoral surveillance networks serve as critical nodes for multidisciplinary intervention strategies.

Taken together, the analysis of the resistome and databases, combined with the implementation of computational pipelines, has enabled us to understand antimicrobial resistance (AMR) not only from a clinical perspective but also within the complex ecological and evolutionary context. New AMR variants emerge from the dynamic interplay among resistance genes (ARGs), mobile genetic elements (MGEs), microbial communities, and environmental factors, including anthropogenic selective pressures. This aligns with the One Health approach, which recognizes the interconnectedness of human, animal, and environmental health within a given ecosystem, as shown in Figure 3 [113].

Therefore, metagenomic approaches will not be limited to a genomic context or to the potential risk of spread. The resistome will be understood as a dynamic system shaped by ecological, clinical, and socioeconomic factors operating at multiple scales.

### 5.1. Antibiotic Use and Exposure

The use of antibiotics remains the primary driver of the spread of ARGs across human, animal, and environmental microbiomes. This is due to the selective pressure exerted by various factors that promote the proliferation of bacteria carrying resistance genes, the horizontal transfer of these determinants among different bacterial species, and the relationships among humans, animals, and the environment within a defined setting in which they interact with one another [114,115].

#### 5.1.1. Impact on the Gut Microbiome

Even short-term exposure to antibiotics can cause profound changes in the composition and function of the gut microbiome. Longitudinal studies have shown significant increases in the relative abundance of ARGs following antibiotic treatment, resulting in decreased microbial diversity [116].

In newborns and preterm infants, prophylactic antibiotic administration is linked to intestinal dysbiosis and an expanded resistome. This effect is mediated by horizontal gene transfer facilitated by MGEs, increasing the risk of colonization by opportunistic pathogens [117]. Bioinformatics tools enable real-time tracking of these events, identifying the co-localization of ARGs with plasmids and other elements through long-read technologies.

#### 5.1.2. Global Variability and Socioeconomic Determinants

Antibiotic use patterns vary significantly across countries and regions, reflecting differences in public health policies, access to medications, and health regulations. This leads to substantial variation in taxonomic and resistance profiles worldwide [118]. Regions with unregulated or excessive antibiotic use show more diverse and widespread resistance, increasing the risk of resistance emerging and spreading [119]. In this regard, the One Health approach is essential for integration not only in the clinical use of antibiotics but also in applications in livestock, aquaculture, and agriculture.

#### 5.1.3. Long-Term Effects and Resistome Memory

One of the most significant findings in recent decades is the long-term persistence of antibiotics’ effects on the resistome. Evidence suggests that early exposure to antibiotics can leave a lasting imprint on the gut microbiota that remains detectable even years later. This imprint includes the persistence of specific ARGs and the stabilization of microbial communities enriched for resistance. Multi-omic approaches and artificial intelligence-based predictive models allow us to begin characterizing and predicting the dynamics of the resistome, opening new perspectives for assessing long-term risks, designing intervention strategies, and understanding the interconnections among ecosystems linked to human, animal, and environmental health [120].

#### 5.1.4. Future Perspectives: Towards a Predictive View of the Resistome

**Figure 4:**
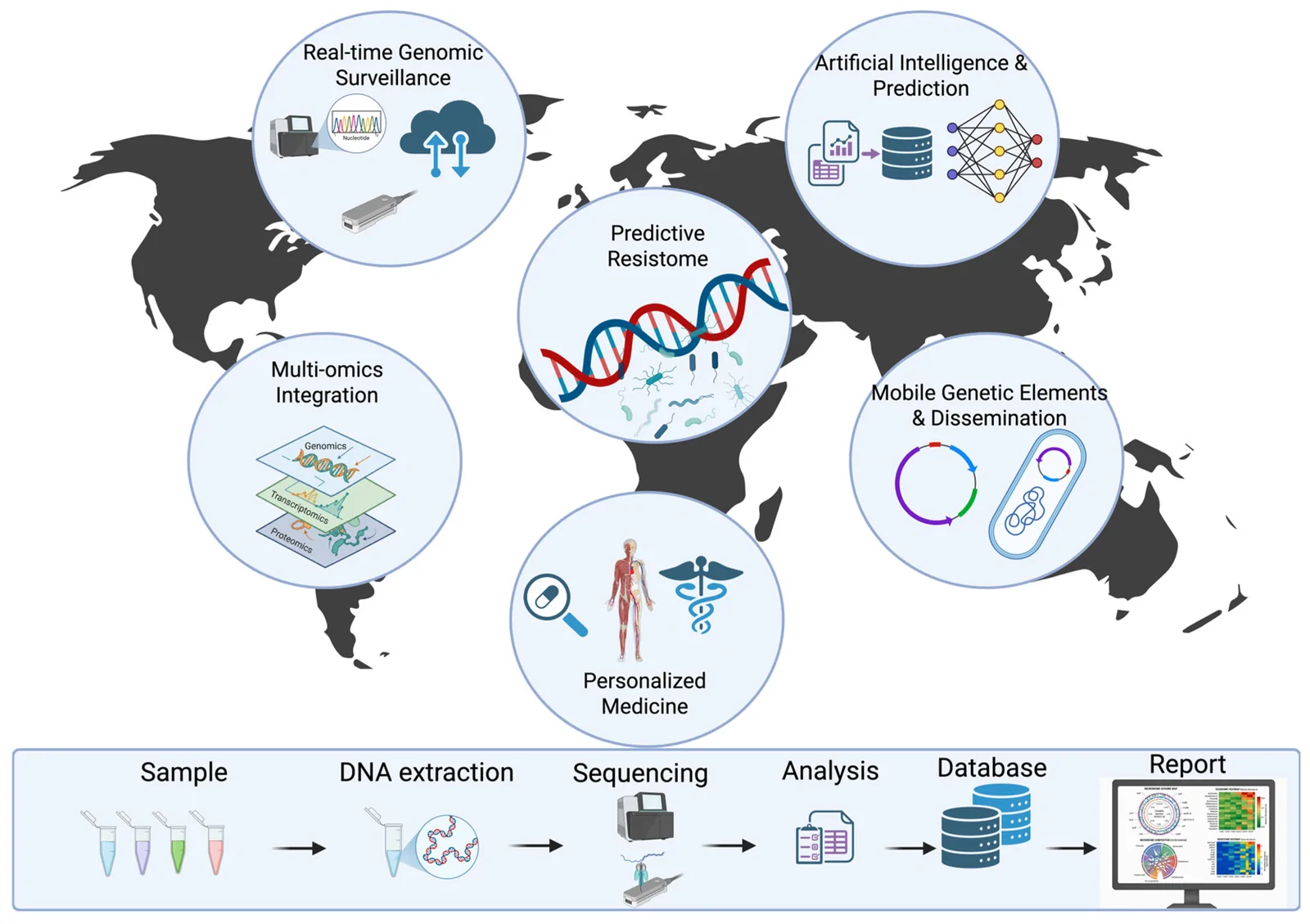
A perspective for studying the global resistome. Genomic surveillance system and personalized medicine. It enables real-time data acquisition and the integration of multi-omics data, including artificial intelligence algorithms that predict the emergence of new ARGs. The diagram illustrates the dynamic feedback between global environmental surveillance and personalized clinical treatment, enabling anticipation of the dissemination of mobile genetic elements within the holobiont and its environment [122].

Integrating metagenomic data with bioinformatics tools and global surveillance platforms will transform the study of the microbiome into a predictive field [121]. These pipelines will not only detect ARGs but also predict their spread using ecological variables, antibiotic usage patterns, and MGE dynamics, as shown in Figure 4.

The future of the field points toward:

- Implementation of real-time surveillance systems based on cloud-based sequencing and analysis.
- Artificial intelligence models capable of predicting the emergence of new resistance mechanisms.
- Global integration of data with other disciplines, such as transcriptomics and proteomics, enables the presentation of information ranging from sequence to structure involved in antibiotic resistance and the synthesis of new drug treatments.
- Development of personalized medicine.

Taken together, these advances establish resistance as a key biomarker for global public health, and a comprehensive study of this phenomenon is essential to mitigating the spread and growth of antimicrobial threats in the coming decades.

### 5.2. Host-Related Factors Shaping the Resistome

The composition of the human microbiome, including its resistome, results from a complex interplay between intrinsic host factors and the environment. Studies show that host genetics is an important factor in shaping the microbiome, but its influence is limited to approximately 2%. Environmental factors largely dominate the structure and function of microbial communities. Among other relevant factors, diet stands out as a major modulator. Dietary patterns influence the intake of macronutrients, micronutrients, fiber, bioactive compounds, and food additives that affect microbial communities [123,124]. Diets rich in ultra-processed foods or containing high levels of residual antibiotics may promote the spread of ARGs, whereas diets high in fiber and a variety of fruits and vegetables have been associated with greater microbial biomass and lower enrichment of resistance genes [125].

Age is another important factor; the gut resistome evolves over a person’s life, reflecting cumulative exposure to drugs, physiological changes, and dietary changes. In early life, particularly during childhood, the resistome is highly dynamic and susceptible to disruption. In adults, it tends to stabilize, although it retains a memory of prior exposures. Older adults exhibit reduced microbial diversity, which may favor the presence of ARGs [126,127].

Changes in the host’s health status associated with dysbiosis have been linked to multiple conditions, including inflammatory bowel diseases, metabolic disorders, liver diseases, and neuropsychiatric disorders [128,129]. These conditions are often accompanied by an expanded resistome, altered microbial structure, and a higher prevalence of opportunistic bacteria. In children with autism spectrum disorders, the microbial profile reflects the close interaction among the microbiome, the immune system, and the nervous system [130,131,132]. Integrating multi-omic approaches with artificial intelligence tools is enabling more precise characterization of the interaction between the host and the pathogen, as well as its neuropsychiatric implications.

### 5.3. Mobile Genetic Elements and Resistome Dissemination

#### 5.3.1. Mechanisms of Horizontal Gene Transfer and Selective Pressure

Mobile genetic elements (MGEs), including plasmids, transposons, integrons, and bacteriophages, are the primary vectors for disseminating antimicrobial resistance genes. These elements facilitate horizontal gene transfer among bacteria, even across phylogenetically distinct species, thereby accelerating the global spread of ARGs [133]. ARGs are often associated with gene regions that contain mobility genes, which facilitate the transfer of new resistance determinants via mechanisms such as conjugation and enable their recombination and accumulation within complex gene platforms [134,135].

The selective pressure from antibiotic use plays a critical role in this context. Sustained exposure to resistant bacteria, along with a higher frequency of horizontal transfer events, promotes the rapid spread of ARGs within and between bacterial species [136]. In environments heavily impacted by human activities, such as hospitals, wastewater treatment systems, and agricultural systems, these processes are significantly intensified [137].

#### 5.3.2. Global Threat and One Health Implications

From a global perspective, MGEs pose a greater threat because they carry critical genes and multidrug resistance determinants such as blaNDM-1, mcr-1, and blaKPC, demonstrating their ability to facilitate the spread of resistance on a global scale [138]. Integrating global data into surveillance platforms has revealed environmental reservoirs of resistance, where MGEs serve as vehicles for long-term persistence and dissemination [139].

From a One Health perspective, the spread of ARGs mediated by MGEs directly links human, animal, and environmental ecosystems [140]. Environmental contamination by antibiotics, heavy metals, and biocides creates constant selective pressure that promotes the spread of these elements. Consequently, controlling antimicrobial resistance requires comprehensive strategies that address all these areas [141,142].

#### 5.3.3. Integrated Perspective on Host-MGE Interactions

Host factors jointly shape resistome dynamics. While the host defines the ecological and physiological context, MGEs act as catalysts for genetic change, facilitating bacterial adaptation to selective pressures across the environments they encounter [143]. Integration at the ecosystem level, along with new bioinformatics and artificial intelligence tools, will enable an understanding of these interactions at unprecedented resolution, which is essential for developing effective global health mitigation strategies [144].

### 5.4. Environmental Reservoirs of the Resistome

The environmental resistome is widely distributed across compartments such as soil, water, and air. These ecosystems serve not only as passive reservoirs but also as dynamic systems that promote the persistence, evolution, and spread of antimicrobial resistance [145]. ARGs circulate among environmental, animal, and human microbiomes, reflecting strong ecological interconnectedness [146].

Soil is one of the oldest and most diverse reservoirs, harboring intrinsic genes that serve as precursors to resistance and can be mobilized against clinical pathogens. In aquatic ecosystems, including rivers, seas, lakes, and wastewater, these reservoirs serve as important pathways for dissemination. These surface water bodies are influenced by anthropogenic activity, which increases the load of ARGs and their diversity [147,148].

Wastewater treatment plants are critical interfaces where microorganisms of human, animal, and environmental origin converge. This environment is conducive to genetic recombination and horizontal transfer of ARGs. Although treatment processes reduce the microbial load, they do not eliminate resistance genes, facilitating their release into the environment and reintegration into the ecological cycle [149,150].

In agricultural systems, antimicrobial use in livestock and aquaculture promotes the selection and spread of ARGs, which are released into the environment through manure and runoff. These ARGs contribute to the contamination of soils and water bodies, facilitating their entry into the food chain and reinforcing the two- way flow between environmental compartments and humans [151,152,153].

Recent advances in ruminant research highlight the profound systemic effects of targeted microbiome modulation on animal production and health. Specifically, dietary supplementation with bioactive agents such as cecropin has been shown to improve growth performance and systemic immunity by modulating rumen fermentation and fibrolytic bacterial communities [154]. Likewise, multi-omics investigations demonstrate that probiotic interventions (*Enterococcus faecalis*) can attenuate metabolic stress, systemic inflammation, and hepatic injury via gut-liver axis crosstalk and arachidonic acid pathways under high-concentrate dietary conditions [155]. Incorporating these livestock-derived mechanistic discoveries strengthens the One Health paradigm by illustrating the fundamental link among animal gut integrity, immune homeostasis, and agricultural sustainability.

Recent evidence shows that the airborne resistome persists in urban and hospital settings, suggesting that air serves as a significant yet underestimated route for the spread of ARGs [156,157]. Taken together, these findings indicate that antimicrobial resistance emerges from complex co-evolutionary processes occurring across highly interconnected systems, in which the environment plays a central role in the generation and persistence of resistance [158].

### 5.5. Epidemiological Surveillance of the Resistome

Epidemiological surveillance of the resistome has shifted from a centralized focus on clinical pathogens to integrated models that include environmental, animal, and human components. This shift addresses the need to tackle antimicrobial resistance on a global scale within the One Health framework [159].

The development of genomic tools, particularly metagenomic sequencing (shotgun metagenomics), has enabled comprehensive characterization of the diversity, abundance, and genetic context of ARGs across various ecosystems. These approaches not only classify known genes but also identify new determinants and assess the potential for mobility, phylogeny, and dissemination of resistance [149].

At the same time, third-generation next-generation sequencing technologies are revolutionizing epidemiological surveillance of the resistome. By enabling whole-genome sequencing and the identification of ARGs within their genetic context, these technologies facilitate the detection and tracking of horizontal transfer events. Sequencing allows resistance genes to be linked to specific hosts, overcoming the inherent limitations of short-read metagenomics [4].

These tools, with their comprehensive approach, enable source-tracking analyses, identify critical reservoirs, and assess the spread of ARGs across different ecological sectors. This is a fundamental strategy for moving toward a predictive surveillance system capable of anticipating emerging risks [160]. However, significant challenges remain, including a lack of standardization in sampling protocols, data analysis, and reporting, which limits comparability between studies and hinders the global integration of information. Therefore, integrating environmental data systems into epidemiological surveillance systems is challenging, particularly in low- and middle-income countries, where the environmental component plays a critical role in exposure to resistance [161].

The outlook will evolve toward integrated real-time surveillance platforms that combine metagenomic sequencing data, antimicrobial resistance profiles, clinical and epidemiological records, and environmental surveillance information through advanced computational and artificial intelligence-based frameworks. In this context, the environmental resistome should be viewed not only as a reservoir but also as an active component of antimicrobial resistance epidemiology. Its systematic integration into One Health surveillance programs will be essential for developing effective strategies to mitigate the global dissemination of antimicrobial resistance.

## 6. Challenges and Future Perspectives

Despite significant progress in characterizing the resistome, limitations remain that hinder its effective integration into epidemiological surveillance and clinical practice. These limitations span methodological, technological, and conceptual aspects, underscoring the need for comprehensive approaches that enable understanding of the dynamics of antibiotic resistance genes (ARGs) in complex, interconnected systems [162].

### 6.1. Standardization, Reproducibility, and Comparability

The lack of standardized protocols for sampling, data processing, and analysis makes it difficult to compare studies and limits reproducibility and standardization across studies. The adoption of frameworks such as STORMS (Strengthening the Organization and reporting of Microbiome Studies) has been proposed to improve transparency and consistency in microbiome studies [163].

The absence of standardized pipelines and computational methods for the identification and quantification of ARGs leads to variable results, depending on databases, algorithms, and models used [164]. This field needs criteria for data normalization and minimum requirements to be met. Other aspects, such as sample size determination in metagenomic studies, can compromise statistical power and the detection of low-abundance ARGs. Tools such as rarefaction curves remain essential for evaluating sequencing depth and resistome coverage [165]. Together, these challenges limit the construction of global comparative frameworks and involvement in integrated surveillance systems [166].

### 6.2. Multi-Omics Integration and Functional Understanding of the Resistome

Although metagenomics has enabled the characterization of the resistome’s composition, significant gaps remain between gene identification and biological functional understanding. Multi-omic integration (metatranscriptomics, metaproteomics, and metabolomics) is essential for characterizing the functional activity of ARGs and their role in microbial ecology [167,168].

One of the main challenges is the transition from genotype to phenotype, since the presence of an ARG does not necessarily imply its expression or clinical relevance. Metatranscriptomics identifies actively expressed genes, while metabolomics can reveal the functional consequences of resistance in microbial ecosystems [169,170].

### 6.3. Technological Advances and Clinical Applications

Next-generation sequencing technologies offer significant advantages, enabling real-time monitoring of circulating strains and the reconstruction of complete genomes, making them particularly useful in clinical and epidemiological surveillance settings [171]. These tools have facilitated the development of rapid, genome-based diagnostic strategies capable of identifying taxonomic and resistance profiles within clinically relevant timeframes. This enables the optimization of antibiotic use, improved decision-making, and the detection of resistance outbreaks [172].

Functional metagenomics remains a key strategy for discovering new ARGs, incorporating the diagnosis of uncultivable pathogens alongside traditional microbiological culture methods, and experimentally validating the functionality of the identified genes [173,174].

In addition to the implementation of artificial intelligence and machine learning, key analytical tools will emerge that enable the processing of large volumes of genomic data, the prediction of resistance mechanisms, and the prioritization of high-risk ARGs based on their mobility and clinical relevance [175,176]. However, their implementation will require robust computational infrastructure and highly trained staff.

### 6.4. Longitudinal Studies and Dynamics of the Resistome

Most current studies on the system are cross-sectional, limiting the ability to infer causal relationships and to understand the temporal dynamics of ARGs. In our view, there is a need to develop longitudinal studies that allow the evaluation of resistome evolution across ecological and clinical contexts [177,178]. Such studies will enable us to understand environmental factors, antibiotic practices, and selective pressures; identify factors associated with the colonization and persistence of resistant bacteria; reduce biases; and improve estimates of prevalence and abundance [179].

### 6.5. Artificial Intelligence and Predictive Surveillance of the Resistome

Resistome analysis faces challenges stemming from the large volume and complexity of data generated by metagenomic sequencing technologies. Traditional approaches based on alignments with reference databases have limitations that can lead to false negatives [180]. Machine learning and deep learning methods are emerging as cutting-edge tools for analysis. These models will enable the identification of complex patterns in genomic data, the classification of resistance profiles, and the prediction of ARG presence without relying exclusively on sequence homologies [181]. In the future, integrating artificial intelligence with multi-omic data and surveillance systems will enable the development of models capable of anticipating emergencies and the spread of antimicrobial resistance. This approach represents a shift toward proactive surveillance, in which the resistome is used as an early indicator of risks in interconnected systems [182].

## 7. Limitations

It is important to acknowledge several methodological limitations inherent in the scope of this review. Although a systematic literature search was conducted in accordance with the PRISMA 2020 guidelines, the rapid evolution and ongoing publication of bioinformatics tools and antibiotic resistance gene (ARG) databases mean that very recent algorithmic frameworks or tools not indexed in the primary databases consulted may have been missed; language restrictions limiting our inclusion criteria to English-language publications may have excluded relevant regional contributions published in other languages; and despite applying predefined eligibility criteria, a degree of qualitative subjectivity persists in selecting and benchmarking specific computational workflows and database structures. Recognizing these limitations provides crucial context for interpreting our synthesis and underscores the need for continuously updated surveillance frameworks.

## 8. Conclusions

The study of the resistome is essential for addressing the growing crisis of antimicrobial resistance. Next-generation sequencing technologies, including long-read sequencing and multi-omics approaches, are expanding our ability to characterize the diversity, evolution, and ecological dynamics of antimicrobial ARGs across human, animal, and environmental systems. However, methodological heterogeneity, the need for standardization, and continuous curation are essential for building more robust and comprehensive ARG databases that enable proper classification and the discovery of important new variants. Future research should focus on standardization, multi-omics integration, optimization of NGS technologies, and the development of specialized centers that enable the storage, management, and analysis of large volumes of biological data, thereby reducing the time required to obtain these results. Due to the extreme dynamism and rapid technological evolution of bioinformatics pipelines and ARG databases, newly emerged algorithmic frameworks released after our final search update may not be fully represented. This will enable longitudinal studies to understand the temporal and functional dynamics of the resistome, strengthen epidemiological surveillance systems, and develop innovative therapies that safeguard the comprehensive concept of health under the One Health approach, promoting interdisciplinary collaboration that recognizes the interconnectedness of human, animal, and environmental health, a critical pathway for sustainably addressing one of the greatest global public health challenges.

## Figures and Tables

**Table 1:** Comparison of molecular approaches for microbiome profiling and resistome characterization.

Method	Primary Objective	Taxonomic Profiling	ARG Detection	Novel ARG Discovery	Genomic Context (ARG-MGE Linkage)	Major Limitations
**qPCR**	Targeted quantification of predefined genes	No	Yes (known ARGs only)	No	No	Limited to selected primer targets; cannot characterize microbial communities or genomic context
**16S rRNA amplicon sequencing**	bacterial and archaeal community profiling	Yes (resolution depends on the targeted variable region, primer design, and database; typically, genus level)	No	No	No	Cannot directly detect or quantify ARGs; limited specie-level resolution and affected by primer bias and 16S copy-number variation
**18S rRNA amplicon sequencing**	Profiling of microbial eukaryotes	Yes (resolution depends on the amplified region and reference database)	No	No	No	Does not characterize bacterial communities or ARGs
**ITS amplicon sequencing**	Fungal community profiling	Yes (species-level resolution often higher than 18S but varies between ITS1 and ITS2 and reference databases)	No	No	No	Restricted to fungal taxonomy; cannot detect ARGs
**Short-read shotgun metagenomics (Illumina)**	Community-wide taxonomic and functional profiling	Yes	Yes	Yes	Limited	Short reads frequently prevent reliable reconstruction of plasmids, integrons, transposons, and other MGEs carrying ARGs
**Long-read shotgun metagenomics (PacBio HiFi/Oxford Nanopore)**	Comprehensive resistome and mobilome characterization	Yes	Yes	Yes	High	Higher DNA input requirements, sequencing cost, and platform-specific error profiles (particularly ONT)
**Functional metagenomics**	Experimental identification of functional ARGs	Partial	Yes	Yes	Functional rather than genomic	Labor-intensive; cloning and heterologous expression bias may prevent recovery of some resistance determinants

**Table 2:** Systematic categorization and comparative analysis of bioinformatics resources, reference databases, and algorithmic frameworks for One Health metagenomic resistome profiling.

Category	Resource/ Tool	Scope & Application	Curation Approach	Key Strengths	Limitations	Maintenance
**1. Core & Specialized ARG Databases**						
**Ontology & clinical ARGs**	CARD [71]	Comprehensive ARG, mutation, and mechanism detection across clinical and environmental taxa	Expert-curated Antibiotic Resistance Ontology (ARO) & RGI models	Mechanistic annotation; captures structural point mutations; gold standard	High computational overhead; risk of false positives from distant homologs	Continuous/Active
	ResFinder [48,72]	Acquired ARG and point mutation identification in clinical isolates and metagenomes	Manually curated database linked to verified phenotypic resistance profiles	High clinical specificity; direct link to phenotypically validated resistance	Underrepresented environmental resistome diversity; limited novel variant detection	Frequent/Active
	AMRFinderPlus [50]	Detection of acquired ARGs, point mutations, stress and virulence genes	Expert-curated by NCBI combining BlastP, HMM profiles, and rules	Integrates mutation and gene detection; direct cross-referencing with NCBI RefSeq	Optimized primarily for refined bacterial assemblies rather than raw reads	Regular (NCBI-driven)
	NDARO	Centralized repository for AMR, virulence, and stress genes across One Health matrices	Curated by NCBI incorporating AMRFinderPlus profiles and Ref Seq genomics	High integration with NCBI GenBank; standardized global surveillance nomenclature	Tailored mainly for assembled bacterial genomes rather than raw metagenomic datasets	Continuous/Active
**Pathogen & Metagenomic Focus**	ARKbase [73]	Integrated AMR knowledgebase covering WHO priority pathogens/ARGs, AST, drug targeted)	Expert-curated integration of genomic, structural and clinical data	Translates pathogen-focused AMR research; integrates ML and drug-target data	Not designed for untargeted metagenomic resistome abundance profiling	Active
	MEGARes [52]	Quantitative metagenomic resistome profiling across complex ecosystems	Manually curated, hierarchical DAG structure (Type, Class, Mechanism, Group)	Optimized for high-throughput short-read alignment; low cross-mapping	Requires strict normalization strategies; less suited for allele-level resolution	Periodic/ Active
	ARGs-OAP [74]	Metagenomic resistome profiling and cell-normalized ARG quantification	Curated sequence repository integrated with the ARGs-OAP pipeline	Specifically optimized for cell-number normalization in environmental samples	Requires specific pipeline structures (ARGs-OAP) for optimal utilization	Periodic
	BACMET [75]	Annotation of antibacterial biocide and heavy metal resistance genes	Expert-curated sequence database linked to experimental literature evidence	Crucial for One Health co-selection dynamics (links biocide/metal resistance to ARGs)	Focused strictly on biocides/metals rather than clinical antibiotic resistance	Periodic
	FARME [53]	Functional metagenomic resistance gene discovery in environmental samples	Curated repository of functionally screened insert libraries from habitats	captures novel uncultured, and non-canonical environmental ARGs	Lacks clinical weight; limited capacity for predicting real-world pathogen spread	Static/Limited
	ARG-ANNOT [51]	Annotation of acquired resistance genes in bacterial genomes	Sequence-based manually curated collection derived from literature	Broad historical ARG coverage; simplified reference structure	Discontinued/infrequent maintenance; superseded by CARD and ResFinder	Legacy/Discontinued
**2. Machine Learning & Novel ARG Discovery**						
**Protein Language Models & AI**	PLM-ARG [76]	Identification of known and highly divergent ARGs from metagenomic data	Pretrained Protein Language Model (PLM) trained on >28,000ARGs	Superior detection of novel/divergent ARGs with low sequence identity	Dependent on training data quality; limited interpretability; lacks regular versioning	Model-dependent
	DeepARG [56]	Deep learning-based prediction of known and novel ARGs from metagenomes	Deep Neural Network (DNN) trained on dissimilarity matrices	High capacity for identifying	High dependency on feature extraction	Model-dependent
	ONN4ARG/HMD-ARG [57,77]	AI-driven sequence classification for novel ARG discovery in metagenomic dark matter	One-class/CNN-hybrid neural networks trained on sequence embeddings	High capacity for identifying uncharacterized ARG families in environmental samples	High dependency on feature extraction quality; requires phenotypic validation	Variable
**3. ARG-Host Attribution & Linkage Methods**						
**Experimental Contextualization**	Native DNA Methylation Profiling [78]	Assigning plasmids and ARGs to bacterial hosts via epigenetic signatures	Bins long read by DNA methylation motifs to identify strain signatures	Enables strain level resolution; links plasmids directly to hosts via epigenetics	Requires high coverage long reads (ONT R10.4); reduced resolution if motifs overlap	Methodological
	Hi-C Proximity Ligation [79]	Linking MGEs and mobile ARGs directly to host genomes in vivo	Covalently crosslinks adjacent DNA intact cells prior to sequencing	Captures physical DNA proximity in vivo; excellent for linking plasmids to hosts	Uneven link density; specialized experimental workflow required	Methodological
	CRISPR-Spacer Mapping [80]	Retrospective host-MGE and host-ARG linkage	Matches bacterial genomic CRISPR spacers against plasmid/MGE backbones	Highly specific when matches exist; provides historical/temporal interaction data	Limited coverage; relies on bacteria possessing active CRISPR systems	Database-dependent
**Computational Binning & Assemblies**	Deep MAG Binners (VAMB, SemiBin2) [81,82]	Genome-resolved metagenomics; placing ARGs into MAGs	Deep variational autoencoders/contrastive self-supervised learning	High organism-level resolution; places ARGs in broader genomic context	Computationally intensive; MGEs and mobile ARGs often fail to bin correctly	Active
	Long-Read Assemblers (hifiasm-meta, metaMDBG, myloasm [83,84,85]	High-contiguity assembly of complete circular MAGs and plasmids	Purpose-built assemblers for PacBio HiFi and Oxford Nanopore reads	Recovers near-complete circular MAGs and full MGEs/plasmids; resolves repeats	Coverage-dependent for rare taxa; higher sequencing cost per base	Active
**4. Mobile Genetic Elements & Contextualization**						
**Mobilome Tracking**	MobileOG-db [86]	Annotation of MGEs linked to horizontal gene transfer (HGT) and resistance	Manually curated database of mobile genetic elements mapped to protein	High-resolution mapping of transposons, integrons, and plasmids; key for HGT risk	Requires integration with separate ARG databases for full genomic context	Periodic/Active
	MOB-suite/PlasFlow/Argo [87,88,89]	Plasmid reconstruction, typing, and read-overlapping ARG-host assignment	Neural networks (PlasFlow)/HMM typing (MOB-suite)/Read overlapping (Argo)	Distinguishes plasmid contigs from chromosomal DNA; enables rapid read linkage	Fragmented assemblies can lead to misclassification of short plasmid contigs	Periodic/Active
**5. Taxonmony & Functional Integration**						
**Taxonomy & Pathways**	GTBD/SILVA/Kraken2/Centrifuge/Perseus [90,91,92]	Standardized taxonomy and ultra-fast k-mer classification	Genome-based marker trees (GTDB)/rRNA alignments/Exact k-mer marching	Gold standard for metagenomic taxonomy; ultra-fast execution (Kraken2)	High memory overhead (GTDB-Tk); sensitive to reference database composition	Active
	KEGG/eggNOG [93,94]	High-level functional, profiling, metabolic pathway mapping, and network inference	Expert-curated biological pathways (KEGG)/Orthologous group mapping (eggNOG)	Enables global functional interpretation and co-occurrence network integration	Proprietary licensing (KEGG); lower manual curation depth in automated orthology	Active

## Data Availability

No new data were created or analyzed in this study. Data sharing is not applicable to this review.

## Abbreviations

The following abbreviations are used in this manuscript:

AI	Artificial Intelligence
AMR	Antimicrobial Resistance
ARO	Antibiotic Resistance Ontology
ARG	Antibiotic Resistance Gene
HGT	Horizontal Gene Transfer
MGEs	Mobile Genetic Elements
NGS	Next-Generation Sequencing
WES	Whole Exome Sequencing
WGS	Whole Genome Sequencing
RPKM/TPM	Reads Per Kilobase Million
